# Colorimetric and Ratiometric Fluorescence Dual-Mode Sensing of Glucose Based on Carbon Quantum Dots and Potential UV/Fluorescence of o-Diaminobenzene

**DOI:** 10.3390/s19030674

**Published:** 2019-02-07

**Authors:** Hong Zhai, Yunfeng Bai, Jun Qin, Feng Feng

**Affiliations:** College of Chemistry and Environmental Engineering, Shanxi Datong University, Datong 037009, China; baiyunfeng1130@126.com (Y.B.); qj187@hotmail.com (J.Q.)

**Keywords:** dual-mode sensor, carbon quantum dots, o-diaminobenzene, glucose

## Abstract

A novel colorimetric and ratiometric fluorescence sensor was constructed by using carbon quantum dots (CQDs) and o-diaminobenzene (ODB). Unlike ODB by itself, ODB oxide (oxODB) not only emits fluorescence, but also produces ultraviolet (UV) absorption. Therefore, on the basis of the potential optical properties of ODB, glucose oxidase (Gox) and horseradish peroxidase (HRP) were introduced into a CQDs–ODB system for the quantitative oxidation of ODB. When glucose is present, it is oxidized by oxygen under the catalytic action of its oxidase to form hydrogen peroxide. Hydrogen peroxide is a strong oxidant that can rapidly oxidize ODB through the catalysis of horseradish peroxidase. oxODB can cause changes in the fluorescence ratio (I_550_/I_446_) and absorbance ratio (A/A_0_). At the same time, the color of the detection solution can also change under sunlight and ultraviolet lamps. Therefore, glucose can be quantitatively detected by ratiometric fluorescence and colorimetry simultaneously, and semi-quantitatively detected by observing the colors with sunlight and ultraviolet lamps of 365 nm. This increases not only the convenience but also the accuracy of detection. In addition, this sensor has good selectivity and can be used for the determination of glucose in serum, providing a new idea for the development of blood glucose sensors.

## 1. Introduction

Diabetes mellitus is a metabolic disease characterized by hyperglycemia, which is caused by insulin secretion defects or insulin dysfunction [1,2]. In addition to high blood glucose, diabetics often have other complications such as tissue damage, blindness, heart disease, stroke and kidney failure. These complications can bring great pain to patients, and some are even disabling and fatal. In order to control or alleviate the disease, patients need to not only take appropriate drugs, but also to regularly determine their blood glucose concentration.

Blood glucose concentration is commonly used as a marker in clinical diagnosis, which is of great significance to the evaluation of a person’s health status [3]. So far, there have been many reports on the determination of blood glucose, including electrochemical analysis [4,5], electrochemiluminescence [6,7,8], chemiluminescence [9,10,11], surface-enhanced Raman scattering [12,13,14], etc. These methods generally have high sensitivity, but usually require specific instruments and equipment, which undoubtedly limits their potential. 

Using colorimetry, which is intuitive and visual, the target content can be directly estimated by observing the color with the naked eye. In view of its advantages, in recent years, colorimetry and other detection methods (e.g., electrochemiluminescence [15] and fluorescence [16]) have been combined to design a number of dual-mode sensors for analysis and detection. Among them, colorimetric and fluorescent dual-mode sensors have attracted much attention, because optical methods can offer many advantages such as high sensitivity, simple instruments and easy operation. For example, Zhao et al. designed a dual-mode protocol for acetylcholinesterase activity and inhibitor screening by using carbon dots and silver nanoparticles as fluorometric and colorimetric reporters, respectively [17]. Furthermore, Wang et al. constructed a dual-mode probe based on phosphorus/nitrogen co-doped carbon quantum dots (CQDs) and gold nanorods for the colorimetric and fluorometric detection of cysteine [18], while Li et al. developed a colorimetric and fluorometric dual-readout sensor based on gold nanoparticles (AuNPs) and graphene quantum dots (SGQDs) for the detection of amifostine (WR2721) [19]. Finally, Priyadarshini et al. prepared Au@carbon dot nanoconjugates and used them as a dual-mode enzyme-free sensing platform for cholesterol [20]. Due to the addition of fluorescence, the colorimetric–fluorescence method not only facilitates the visualization of target detection, but also provides high sensitivity, which can better meet the requirements of analysis.

With the advent of ratiometric fluorescence detection, a series of dual-mode sensors have been designed based on colorimetry and the ratiometric fluorescence method. Such sensors are not only intuitive and sensitive, but also more accurate. Because the ratiometric fluorescence method detects the ratio of fluorescence intensity of two fluorophores, it can reduce or even eliminate errors caused by changes in the environment, instruments and parameters. Kuo et al. prepared polydiacetylene -functionalized polymer dots for the colorimetric and fluorescent ratiometric detection of lead ions in living cells [21]. Fang et al., on the other hand, developed a ratiometric fluorescence and colorimetric method based on Yb^3+^, Er^3+^ and Tm^3+^ co-doped NaYF_4_ nanoparticles and ZnFe_2_O_4_ magnetic nanoparticles for the detection of uric acid with satisfactory results [22]. Chen et al. fabricated a bimetallic metal–organic framework (MOF) with a yellow emission signal and chose this MOF as a ratiometric fluorescence and colorimetric sensor for methanol, both in solution and vapor states [23]. Finally, Feng et al. used iminocoumarin as a new colorimetric and ratiometric fluorescent probe for the rapid detection of the highly toxic phosgene with high selectivity and sensitivity [24]. However, few reports have been made on colorimetric and ratiometric fluorescent dual-mode glucose sensing. Therefore, it is necessary to develop such sensors for glucose detection.

In this study, a colorimetric and ratiometric fluorescent dual-mode glucose sensor was designed based on the optical properties of o-diaminobenzene oxide (oxODB) for two reasons: (1) oxODB can produce ultraviolet (UV) absorption, which is used as the reporter of colorimetry for glucose detection; (2) oxODB can produce fluorescence, and can also quench the fluorescence of CQDs. Hence, the fluorescence intensity ratio of oxODB to CQDs can be used as the reporter of the ratiometric fluorescence method for the detection of glucose. oxODB is obtained by two successive biological oxidation reactions. Specifically, ODB is oxidized by hydrogen peroxide in the presence of horseradish peroxidase (HRP), and hydrogen peroxide is obtained by the oxidation of glucose in the presence of its oxidase. Therefore, glucose can determine the generation of oxODB, and oxODB determines the changes of absorbance and fluorescence ratio simultaneously, so glucose can be detected in two ways by this method. In addition, the color of the glucose detection solution can show significant gradient change under sunlight and ultraviolet lamps, so a crude determination of the glucose content can be performed directly with the naked eye.

## 2. Materials and Methods

### 2.1. Chemicals and Apparatus

Glucose and glucose oxidase (Gox) were received from Sigma-Aldrich (St. Louis, MO, USA). o-Diaminobenzene (ODB), horseradish peroxidase (HRP), fructose, sucrose, maltose, xylose, lactose and galactose were purchased from Aladdin Biochemical Technology Co., Ltd. (Shanghai, China). Citric acid and ethylenediamine were obtained from Beijing Chemical Works (Beijing, China) and Tianda Chemical Reagent Factory (Dongli, Tianjin, China), respectively. Normal human serums were purchased from Beijing Solarbio Science and Technology Co., Ltd. (Beijing, China). Ultrapure water (≥18.2 MΩ·cm) was used throughout all experiments. All reagents were obtained from commercial suppliers and used without further purification.

Fluorescence measurements were collected using a Perkin Elmer LS-55 (Waltham, MA, USA) spectrometer, equipped with a quartz cuvette with a 1-cm path length. A TEM image of CQDs was recorded using a Tecnai F20 transmission electron microscope (FEI) operated at a voltage of 200 kV (Hillsboro, OR, USA). UV-vis absorption spectra were obtained using a Perkin Elmer Lambda 35 UV/vis spectrometer (Waltham, MA, USA) with a quartz cuvette with a 1-cm path length.

### 2.2. Synthesis of Fluorescent CQDs

CQDs were prepared according to the microwave-assisted hydrothermal method [25] with some modifications. A total of 1.6 mg citric acid, 40 mL water and 2 mL ethylenediamine were added to the beaker successively and stirred until dissolved. Then, the mixture was put in a microwave oven at 750 W for 10 min to obtain a brownish red substance. Finally, water was added to dissolve the brownish red substance and centrifuged. The supernatant was dialyzed in pure water for 3 days and fluorescence CQDs were obtained.

### 2.3. Development of a Colorimetric and Ratiometric Fluorescence Dual-Mode Sensor for the Detection of Glucose

A series of different concentrations of glucose solution (100 μL) and 10 μL Gox (2 mg/mL) were mixed and reacted for 30 min. Then, 2 μL HRP (1 mg/mL), 5 μL ODB and 10 μL CQDs (1 mg/mL) were added to the mixture and the final volume reached 500 μL with 0.01 mol/L PBS buffer. After reaction for a period of time, fluorescence and absorption spectra were collected separately. 

### 2.4. Selectivity Experiment

Glucose structure analogues (sucrose, maltose, fructose, xylose, lactose and galactose) were taken to determine the fluorescence and absorption spectra according to the experimental steps in Section 2.3, and the final concentration of each analogue was 2 mM.

### 2.5. Determination and Spiking Recoveries of Glucose in Normal Human Serum

The serum samples were subjected to a simple pretreatment: 1 mL of sample was precipitated with the equal volume of acetonitrile and centrifuged at the speed of 13,000 rpm for 10 min. The supernatant was transferred to another centrifuge tube, dried with nitrogen and then dissolved in 5.0 mL PBS buffer solution (pH 7.0). A total of 30 μL of treated serums was applied to the detection system described in Section 2.3 for the determination of glucose.

A certain amount of glucose solution was added to the serum sample to bring the spiking level up to 5 mM, and then the glucose concentration was determined according to the above steps. The spiking level was assayed five times.

## 3. Results

### 3.1. The Principle of a Dual-Mode Sensor for Detecting Glucose 

**Figure 1:**
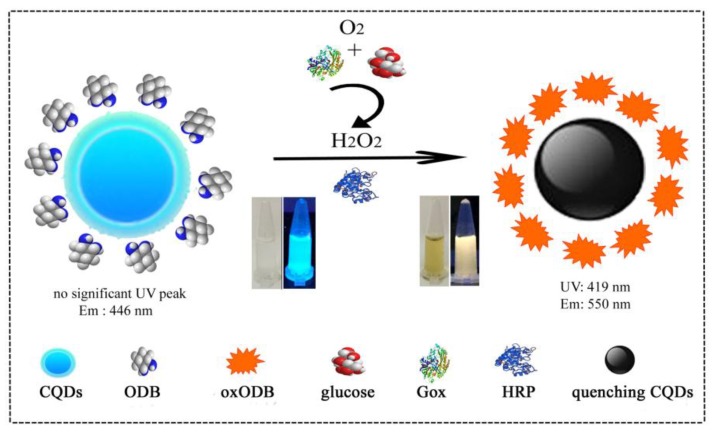
The detection principle of a dual-mode glucose sensor based on carbon quantum dots (CQDs) and o-diaminobenzene (ODB).

ODB is a strong reductant that can be oxidized by oxygen or hydrogen peroxide. The oxidation product can emit fluorescence when excited by light with 380 nm. In addition, ODB oxide (oxODB) is an electron-deficient molecule that can be used as an effective quencher for CQDs. On the basis of the optical characteristics of oxODB, glucose oxidation reaction and HRP were introduced into a CQDs–ODB system to develop a dual-mode sensor for the determination of glucose. The detection principle is shown in Figure 1. Glucose was oxidized to produce gluconic acid and hydrogen peroxide under the catalytic action of glucose oxidase. Hydrogen peroxide, as an oxidant, was able to rapidly oxidize ODB with the action of horseradish peroxidase. oxODB produced a fluorescence peak at 550 nm, and the intensity increased with glucose. Meanwhile, oxODB was able to quench the fluorescence of CQDs, and the quenching degree gradually increased with oxODB; that is, the fluorescence of CQDs decreased with the increase of glucose. Thus, glucose was able to cause fluorescence changes in CQDs and oxODB. In order to reduce or eliminate errors caused by the external environment and instrument parameters, the ratio of the fluorescence intensity of oxODB to CQDs was used as the detection signal to detect glucose. At the same time, the fluorescence color of the test solution also changed significantly with the increase of the glucose concentration. Therefore, the glucose content could be estimated by directly observing the fluorescence color. 

In addition, oxODB was able to produce an ultraviolet absorption peak, and the absorbance at 419 nm and color regularly changed with glucose content, so colorimetry could also be used to detect glucose.

### 3.2. Characterization of CQDs

**Figure 2:**
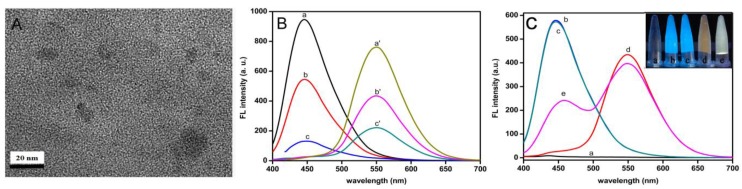
(**A**) The TEM of CQDs. (**B**) Fluorescence spectra of CQDs and ODB oxide (oxODB) at different excitation wavelengths (a, c′:365 nm; b, b′:380 nm; c, a′:400 nm). (**C**) Fluorescence spectra of CQDs, ODB, oxODB and their related mixtures (a: ODB, b: CQDs, c: CQDs + ODB, d: oxODB, e: CQDs + oxODB). oxODB is the product of the oxidation of ODB by hydrogen peroxide (the oxidation product of glucose) under horseradish peroxidase (HRP); the insert images are the corresponding fluorescence color under ultraviolet (UV) lamps of 365 nm.

CQDs were prepared from citric acid and ethylenediamine through microwave heating. As can be seen from the TEM image (Figure 2A), CQDs had a good dispersion but a certain degree of aggregation; they were able to produce a strong emission peak at 446 nm. Moreover, as the excitation wavelength increased, its fluorescence intensity gradually decreased (Figure 2B). ODB itself did not fluoresce and did not produce any emission peak, as shown in Figure 2C (curve a). However, its oxide (oxODB) was able to produce a strong emission peak at 550 nm. Meanwhile, the fluorescence intensity of oxODB gradually increased with the excitation wavelength (Figure 2B). By comparing the fluorescence spectra of CQDs and oxODB at different excitation wavelengths, it was found that the changes of their fluorescence intensity were exactly the opposite. Therefore, we chose 380 nm as the fluorescence excitation wavelength.

Figure 2C shows the fluorescence spectra of CQDs, ODB, oxODB and the relevant mixtures. After ODB was mixed with CQDs, the spectrum of the mixture was basically the same as that of CQDs (curves b and c), indicating that ODB had a weak quenching effect on CQDs. Curve d is the fluorescence spectrum of ODB oxidized by hydrogen peroxide (generated from the oxidation of glucose) under the catalysis of HRP, and its shape is the same as that of oxODB in Figure 2B. Curve e refers to the spectrum corresponding to the determination of 200 μM glucose with the proposed method. Although the fluorescence of CQDs decreased at 446 nm, a new fluorescence peak of oxODB was generated at 550 nm. Therefore, this method could detect glucose by the fluorescence ratio of oxODB and CQDs. 

Apart from emitting fluorescence, oxODB was also able to produce ultraviolet (UV) absorption at 419 nm (see curve d in Figure S4), whereas ODB by itself could not (curve c). In addition, CQDs (0.02 mg/mL) in the detection system had no obvious absorption (curve a), although a high concentration of CQDs was able to produce a UV peak at 350 nm (curve b). This meant that the existence of CQDs did not interfere with the determination of glucose by oxODB absorbance. Curve e is the UV spectrum of the glucose detection solution, the shape of which is the same as that of oxODB (curve d), further proving that glucose could be measured by oxODB absorbance. This means that glucose could be measured by colorimetry.

### 3.3. Optimization of Main Experimental Parameters

In order to obtain the best detection results, the main experimental parameters were optimized, including ODB concentration and ODB oxidation time.

**Figure 3:**
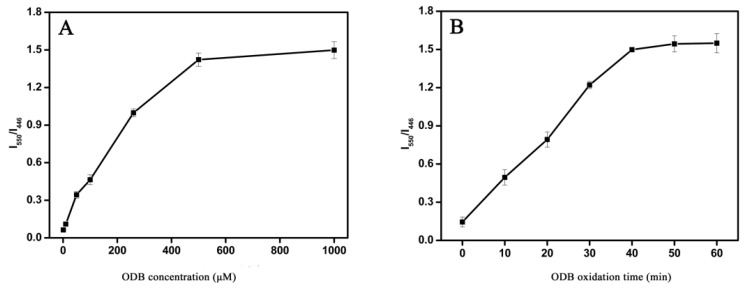
Optimization of main experimental parameters: (**A**) The effect of ODB concentration on the fluorescence ratio (I_550_/I_446_); the glucose concentration is 200 μM. (**B**) The effect of ODB oxidation time on the fluorescence ratio (I_550_/I_446_); the concentrations of glucose and ODB are 200 μM and 500 μM, respectively.

As a potential fluorescer, the concentration of ODB directly determined the fluorescence intensity of oxODB, and was related to the degree of CQDs fluorescence quenching. Therefore, the concentration of ODB affected the fluorescence ratio of oxODB to CQDs (I_550_/I_446_). As can be seen from Figure 3A, because of the increasing ODB concentration, I_550_/I_446_ gradually increased first and then remained unchanged. The oxidation product hydrogen peroxide was also fixed for a certain amount of glucose. Therefore, because of the increase of ODB concentration, the generated oxODB and fluorescence intensity gradually increased as well. At the same time, the degree of fluorescence quenching of CQDs also increased, resulting in the gradual increase of I_550_/I_446_. However, when ODB was excessive, the amount of oxODB did not continue to increase, because hydrogen peroxide had been used up; this kept the fluorescence of oxODB, CQDs and ultimately I_550_/I_446_ unchanged. Therefore, we chose 500 μM as the optimal concentration of ODB.

ODB oxidation time was another important parameter for glucose detection, and the results are shown in Figure 3B. As the reaction time increased, I_550_/I_446_ gradually increased and then remained unchanged. At the beginning, as oxODB increased continuously over time, the fluorescence of oxODB and its effect on CQDs fluorescence were gradually enhanced, leading to the gradual increase in I_550_/I_446_. However, after 40 min, as the ODB had been completely oxidized, the amount of oxODB could not change anymore, which resulted in the value of I_550_/I_446_ remaining the same. Therefore, it was decided that the optimal oxidation time of ODB is 40 min.

### 3.4. Detection of Glucose Using the Dual-Mode Sensor

**Figure 4:**
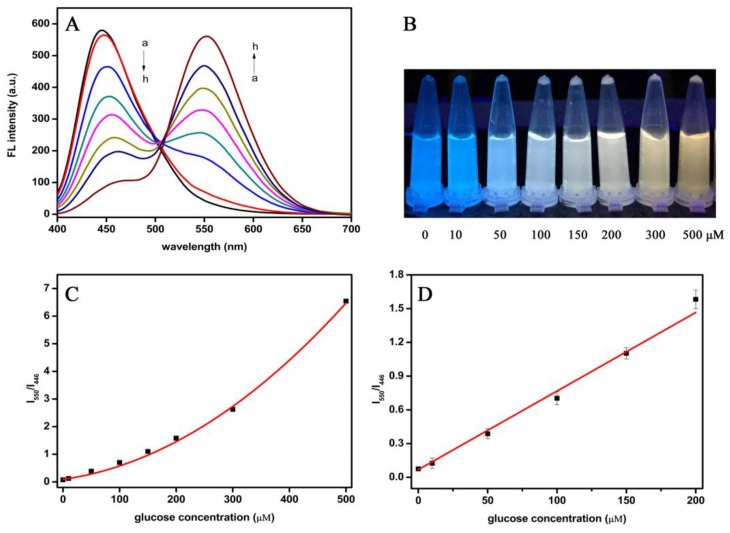
(**A**) The fluorescence spectra for detecting glucose with different concentrations (a→h, the final glucose concentrations are 0, 10, 50, 100, 150, 200, 300, 500 μM, respectively) using the dual-mode sensor. (**B**) The colors of glucose detection solutions at different concentrations under the ultraviolet light of 365 nm. (**C**) The relationship curve between the fluorescence ratios of oxODB to CQDs (F_550_/F_446_) and glucose concentrations between 0 and 500 μM. (**D**) The linear relationship between I_550_/I_446_ and glucose concentrations ranging from 0 to 200 μM.

A series of glucose standard solutions with different concentrations were detected by the dual-mode sensor (results are shown in Figure 4). Because of the increase in glucose concentration, the fluorescence peak of CQDs at 446 nm decreased gradually. On the contrary, the peak of oxODB at 550 nm kept increasing (Figure 4A). In order to establish the quantitative relationship between the fluorescence changes of CQDs and oxODB and glucose concentration, we linearly fitted the fluorescence ratio of oxODB and CQDs (I_550_/I_446_) to glucose concentration (results are shown in Figure 4D). The fluorescence ratio (I_550_/I_446_) showed a good linear relationship with glucose in the concentration range of 10~200 μM. The linear equation was y = 0.00698x + 0.0682 and the correlation coefficient R^2^ was 0.9933. The detection limit was calculated according to the formula (3σ/s), and the value was 1.15 μM (where σ is the standard deviation of 11 blank solutions and s is the slope of the regression equation). Moreover, the color of the glucose detection solution showed significant gradient change under UV lamps of 365 nm, as shown in Figure 4B. Because of their different colors, we could directly estimate their glucose content with the naked eye, which can be very convenient for detection. Compared with other fluorescence detection methods reported for glucose (Table S1), the detection limit of this method was far lower than that of literatures [26,27]. It is consistent with that of the literatures [28,29], which indicates that it has good sensitivity.

In addition, we also performed UV spectroscopy for the determination of glucose at different concentrations, and the results are shown in Figure S5. Because of the increase in glucose concentration, the absorbance at 419 nm increased continuously. After linear fitting, the absorbance ratio (A/A_0_, where A and A_0_ represent the absorbance at 419 nm in the presence and absence of glucose) and glucose concentrations showed a good linear relationship between 10 μM and 500 μM. The linear equation was y = 0.4058x + 0.9882, the correlation coefficient R^2^ was 0.9944 and the detection limit was 3.00 μM. Furthermore, the colors of the detection solution also showed gradient change under sunlight, and the glucose content could be roughly determined based on color depth (Figure S5). Compared with other colorimetric methods (Table S2), the detection limit of this method was far lower than that of literatures [30,31] and was the same as that of literatures [32,33].

In summary, glucose was quantified simultaneously by a fluorescence ratio (I_550_/I_446_) and an absorbance ratio (A/A_0_), which greatly increased the accuracy of detection. Moreover, the semi-quantitative determination of glucose was also carried out through the use of ultraviolet lamps and sunlight, by directly observing the color of the detection solution; this greatly increased the convenience of the method, the applicable scope of which could be expanded to benefit more people.

### 3.5. The Selectivity of the Dual-Mode Sensor

**Figure 5:**
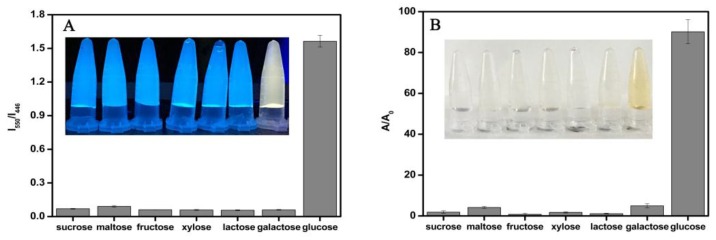
The interference experiments of glucose structure analogues to glucose using: (**A**) Ratiometric fluorescence and (**B**) a colorimetric method (the interferents’ concentration is 2 mM; the glucose concentration is 200 μM).

Certain types of sugar with a similar structure to glucose can sometimes interfere with its determination. Hence, we selected six glucose structure analogues (fructose, xylose, galactose, lactose, sucrose and maltose) as potential interferences to determine the selectivity of this method, and the results are shown in Figure 5. When the interferents were detected, their concentrations were all higher than glucose (10 times higher than the glucose concentration), but their fluorescence ratios (I_550_/I_446_) and absorbance ratios (A/A_0_) were very small. Because glucose oxidase was highly specific, it could only catalytically oxidize glucose to produce hydrogen peroxide, which then oxidized ODB and caused significant changes in the fluorescence and absorbance ratios. Therefore, this method had good selectivity.

### 3.6. Real Samples Detection

In order to verify the practicability of this method, we selected two samples of normal human serum to determine their glucose levels, and the results are shown in Table 1. The glucose levels were 4.48 mM and 5.33 mM, respectively, while their relative standard deviations were 5.4% and 6.1%; these were in accordance with commercial glucose kits (4.51 mM and 5.35 mM). In order to further verify the reliability of the method, we performed a spiking recovery test, and the recoveries of the two samples were 105.6% and 105.0%, respectively, with relative standard deviations of 4.3% and 5.7%. Therefore, this method has strong practicability and can be used for the determination of real serum samples.

### 3.7. Preparation of a Glucose Test Paper Based on the Dual-Mode Sensor

**Figure 6:**
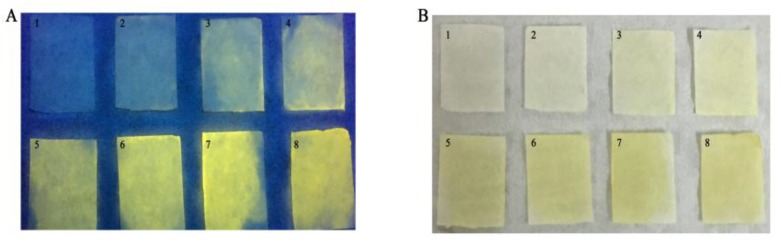
(**A**) Color changes of test papers impregnated with glucose oxidase (Gox), HRP, ODB and CQDs under 365 nm UV light, after immersion into solutions containing 0, 10, 50, 100, 150, 200, 300 and 500 μM glucose, respectively. (**B**) Their corresponding color changes under sunlight.

To further apply this sensor for on-site detection, we prepared a test paper for glucose. The steps were as follows: First, we prepared a mixture solution containing Gox, HRP, ODB and CQDs, and then immersed the filter papers in the mixture for 10 min. After drying, we immersed the filter papers into the target solutions containing glucose for 40 min. As shown in Figure 6, the color under both the UV light and sunlight showed significant gradient changes, which were consistent with the spectral response. The results demonstrated that the dual-mode sensor has promising potential for on-site visual detection of glucose.

## 4. Conclusions

In this study, on the basis of the different optical properties of ODB and its oxide (oxODB), glucose oxidase and horseradish peroxidase were introduced into a CQDs–ODB system to construct a novel colorimetric–fluorescence ratio dual-mode sensor for glucose detection. In the presence of glucose, ODB was oxidized and converted into oxODB after two successive oxidation reactions. oxODB caused changes to the fluorescence ratio (I_550_/I_446_) and the absorbance ratio (A/A_0_). At the same time, the color of the detection solution also changed under sunlight and ultraviolet lamps of 365 nm. Therefore, this method can simultaneously perform fluorescence and ultraviolet determination of glucose to achieve quantitative detection, and achieve semi-quantitative detection by observing the color of the detection solution under sunlight and ultraviolet light. This increases not only the convenience of detection, but also its accuracy. In addition, experimental results showed that the sensor has good selectivity and can be used to detect glucose in normal serum with satisfactory results. In conclusion, the sensor has the advantages of high specificity, high accuracy and intuitiveness, which can provide a new idea for glucose detection.

## Figures and Tables

**Table 1:** The results of spiking recoveries of glucose in the samples of normal human serum.

Sample	Added (mM)	Found (mM)	Recovery (%)	RSD (%)
1	0	4.48	-	-
	5	9.76	105.6	4.3
2	0	5.33	-	-
	5	10.58	105.0	5.7

## Supplementary Materials

The following are available online at http://www.mdpi.com/1424-8220/19/3/674/s1. Figure S1: Particle size distribution of CQDs; Figure S2: The zeta potential of CQDs; Figure S3: The stability experiment of CQDs in PBS; Figure S4: Absorption spectra of CQDs, ODB, oxODB and their related mixtures; Figure S5: The detection of glucose with different concentrations by use of the colorimetric method in a dual-mode sensor. Figure S6: CQDs fluorescence spectra in the reaction medium at different times; Table S1: Comparison of reported fluorescence methods and the proposed method for glucose; Table S2: Comparison of reported colorimetric methods and the proposed method for glucose.
