# Supplementary material for: Colorimetric and Ratiometric Fluorescence Dual-Mode Sensing of Glucose Based on Carbon Quantum Dots and Potential UV/Fluorescence of o-Diaminobenzene
Source: Sensors (Basel). 2019 Feb 7;19(3):674. doi: 10.3390/s19030674 (PMC6386939; doi:10.3390/s19030674)
Supplement: Supplementary file 1 [file sensors-19-00674-s001.pdf]

Electronic Supporting Information

Colorimetric and Ratiometric Fluorescence Dual Mode Sensing of Glucose Based on Carbon Quantum Dots and Potential UV/Fluorescence of o-Diaminobenzene

Hong Zhai*, Yunfeng Bai, Jun Qin, Feng Feng*

College of Chemistry and Environmental Engineering, Shanxi Datong University, Datong 037009, China;

baiyunfeng1130@126.com (Y.F.B.); qj187@hotmail.com (J.Q.)

***** Correspondence: zhhong81@126.com (H.Z.); feng-feng64@263.net (F.F.), Tel: +86-352-7158662

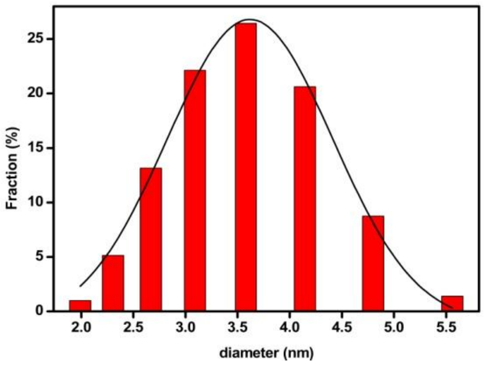

Figure S1 Particle size distribution of CQDs

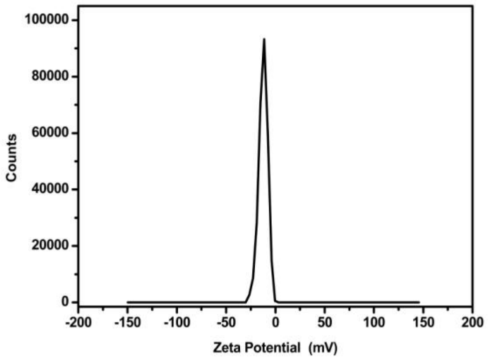

Figure S2 The zeta potential of CQDs

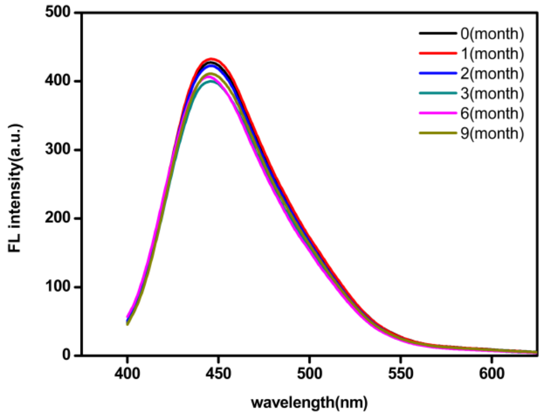

Figure S3 The stability experiment of CQDs in PBS

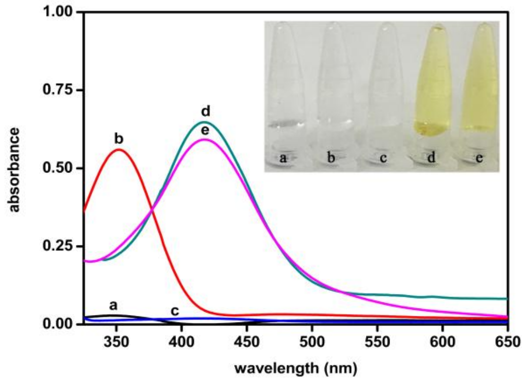

Figure 4 Absorption spectra of (a) 0.02 mg/ ml CQDs, (b) 2 mg/ml CQDs, (c) 1mM ODB, (d) oxODB, (e) 200 μM glucose detection solution.

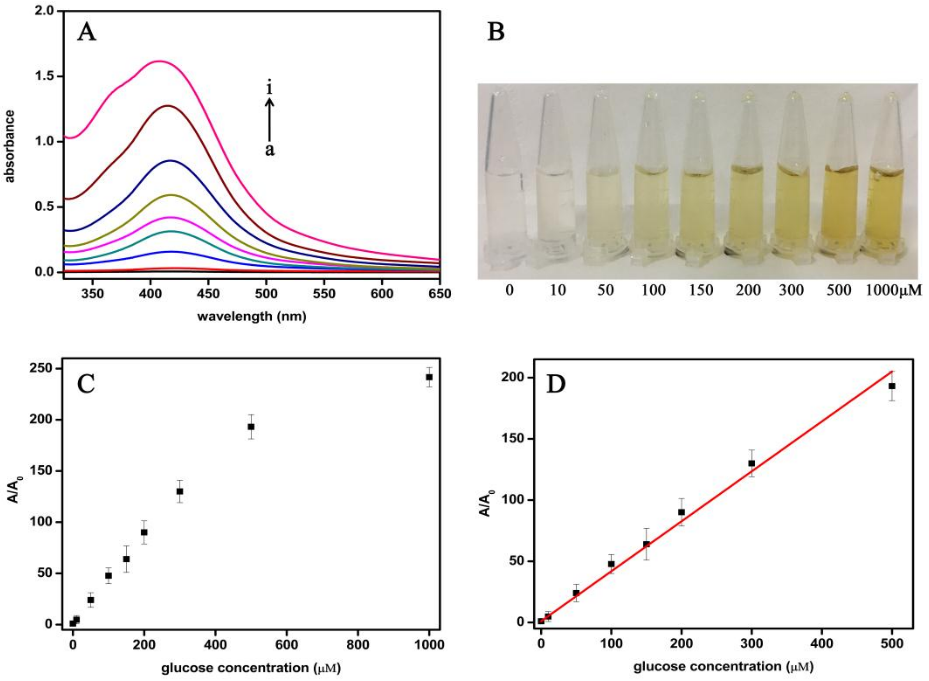

Figure S5 (A) Absorbance spectra for the detection of glucose with different concentrations. (B) The colors of glucose detection solution with different concentrations. (C) The relationship curve between absorbance ratio (A/A_0) and glucose concentration between 10 and 1000 μM. (D) The linear relationship between A/A_0 and glucose concentrations ranging from 10 to 500 μM.

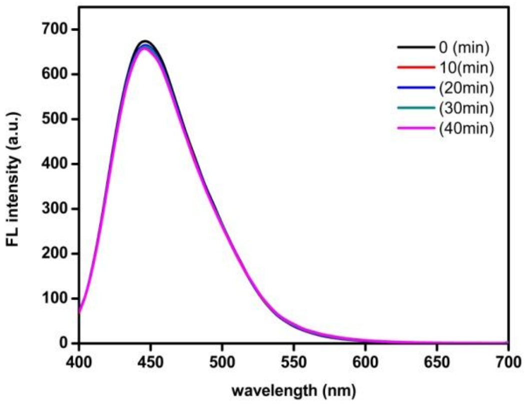

Figure S6 CQDs (0.02 mg/ml) fluorescence spectra in the reaction medium at different times

Table S1 Comparison of recently reported fluorescence methods for the detection of glucose

Material	Method	Linear range	Detection limit	Reference
B-doped carbon quantum dots (BCQDs)	fluorescence	8～80 μM	8 μM	[26]
CdTe/CdS quantum dots–glucose oxidase complex	fluorescence	1～7 mM	0.1 mM	[27]
Ag@CDs composite	fluorescence	7～20μM	1.55μM	[28]
C-dots/AgNPs	fluorescence	2～100μM	1.39 μM	[29]
CDs-ODB	fluorescence	10～200 μM	1.15μM	This Work

Table S2 Comparison of recently reported colorimetric methods for the detection of glucose

Material	Method	Linear range	Detection limit	Reference
Ag nanoparticles/ cotton fabric	colorimetry	0.1～2 mM	0.08 mM	[30]
chloranil	colorimetry	0.03～1 mM	0.03 mM	[31]
MoS_2@$MgFe_2O_4$ nanocomposite	colorimetry	5～200μ M	2.0 μM	[32]
Fe_3O_4@Au-Cys-FA-GOx nanocomposites	colorimetry	10μM～1mM	3.8μM	[33]
CDs-ODB	colorimetry	10～500 μM	3 μM	This Work
